# Association Between Food Environment Characteristics and Overweight and Anemia in Socially Vulnerable Children Living in Favelas

**DOI:** 10.3390/ijerph23060801

**Published:** 2026-06-15

**Authors:** Luiz Gonzaga Ribeiro Silva-Neto, Risia Cristina Egito de Menezes, Vanessa Sá Leal, Thays Lane Ferreira dos Santos, Telma Maria de Menezes Toledo Florêncio

**Affiliations:** 1 Programa de Pós-Graduação em Nutrição, Escola Paulista de Medicina, Universidade Federal de São Paulo, São Paulo 04023-062, Brazil; 2 Programa de Pós-Graduação em Nutrição, Faculdade de Nutrição, Universidade Federal de Alagoas, Maceió 57072-970, Brazil; 3 Curso de Nutrição, Centro Acadêmico de Vitória, Universidade Federal de Pernambuco, Vitória de Santo Antão 55608-680, Brazil

**Keywords:** food environment, ultra-processed beverages, overweight, anemia, children

## Abstract

**Highlights:**

**Public health relevance—How does this work relate to a public health issue?**

- This study examines how overweight and anemia coexist among children in socially vulnerable communities, emphasizing that both are significant public health issues disproportionately impacting low-income populations.
- The findings highlight that unhealthy food environments, with high availability of ultra-processed beverages and limited access to fresh foods, are related to childhood malnutrition in its multiple forms.

**Public health significance—Why is this work of significance to public health?**

- The findings indicate that greater availability of fruits and vegetables in the food environment is associated with a lower chance of overweight, whereas higher availability of ultra-processed beverages is associated with an increased chance of overweight and anemia in children.
- By focusing on children living in favelas and urban communities in Northeastern Brazil, this research provides important data on vulnerable populations that are often underrepresented in food environment studies.

**Public health implications—What are the key implications or messages for practitioners, policy makers and/or researchers in public health?**

- To address these issues, public policies should promote and increase the availability of fruits, vegetables, and fresh foods, and reduce children’s exposure to ultra-processed beverages in socially vulnerable areas to mitigate overweight and anemia.
- Practitioners and researchers should target the food environment in interventions to prevent both childhood overweight and micronutrient deficiencies, especially anemia, focusing on settings most at risk.

**Abstract:**

Background: The food environment plays a significant role in determining children’s nutritional status, especially in socially vulnerable settings, where the high availability of ultra-processed beverages can contribute to both overweight and nutritional deficiencies, such as anemia. Thus, this study aimed to assess the association between the availability of fruits, vegetables, and ultra-processed beverages in the food environment and the occurrence of overweight and anemia in children living in socially vulnerable areas. Methods: This is a cross-sectional study with an ecological component, conducted between 2020 and 2021, involving 665 children residing in 40 favelas and urban communities in Maceió, Alagoas, Brazil. Socioeconomic, anthropometric, and hematological data were collected, along with a characterization of the food environment in 624 establishments using the AUDITNOVA tool, focusing on the availability of fruits, vegetables, and ultra-processed beverages. The outcomes investigated were overweight (BMI-for-age z-score > +2) and anemia (hemoglobin < 11 g/dL). Multilevel models were used to assess the associations between the food environment and the outcomes of interest. Results: The prevalence of overweight was 19.7%, while anemia affected 50.4% of the children assessed. Greater availability of fruits and vegetables was associated with a lower chance of being overweight (OR: 0.82; 95% CI: 0.79–0.98). In contrast, high availability of ultra-processed beverages was associated with a higher chance of being overweight (OR: 1.35; 95% CI: 1.07–1.84) and anemia (OR: 1.53; 95% CI: 1.04–2.29). Conclusion: Food environments characterized by widespread availability of ultra-processed beverages were associated with a higher prevalence of overweight and anemia among children. In comparison, greater availability of fresh or minimally processed foods was associated with a lower prevalence of overweight. These findings highlight the importance of public policies that promote healthier food environments in socially vulnerable areas.

## 1. Introduction

The food environment is the space where food is accessed, serving as the interface between consumers and food systems. Its understanding requires consideration of characteristics such as convenience, availability, and physical and financial accessibility to foods [1], as well as the multiple contexts in which foods are acquired. However, it is important to recognize its diversity, particularly in low- and middle-income countries such as Brazil, given that malnutrition is related to individuals’ exposure to the foods available in the surroundings of their homes and in the places they routinely circulate [1,2].

In this context, assessing the consumer food environment becomes particularly relevant. In Brazil, for example, food availability varies according to the region’s vulnerability, with low availability of fresh and minimally processed foods and a high prevalence of ultra-processed foods (UPFs) commonly observed in areas with greater social vulnerability [3,4].

The widespread availability of UPFs in the environment [5,6] has been associated with dietary patterns characterized by greater consumption of these products and with overweight and nutritional deficiencies, such as anemia, during childhood [7]. In this regard, changes in childhood nutritional status, especially among populations experiencing greater social vulnerability [8], are influenced by multiple factors, including dietary patterns [9], infectious diseases [10], sanitation conditions [11], physical activity [12], and socioeconomic circumstances [13]. Among these determinants, dietary patterns characterized by increased consumption of UPFs [7,14], particularly ultra-processed beverages [15,16], have received increasing attention in the literature. This scenario is even more concerning given the reduced consumption of fresh foods, such as fruits and vegetables [17].

Thus, it is evident that the food environment may influence the quality of children’s diets and food choices, with potential implications for nutritional status [18,19]. Nevertheless, further investigation is needed to better understand how the availability of specific foods, including fruits, vegetables, and ultra-processed beverages, relates to children’s nutritional status, as this information is essential for guiding interventions, particularly among more vulnerable populations. Furthermore, associations observed between environmental characteristics and health outcomes should be interpreted alongside evidence from diverse methodological approaches and related determinants of health, thereby strengthening the evidence base regarding the role of food environments in nutritional outcomes.

Considering the contextual nature of food environment measures, the interpretation of associations between environmental characteristics and individual health outcomes benefits from integration with evidence on individual and other contextual determinants of health. To better understand overweight and anemia, which are highly prevalent among children in impoverished areas, this study aimed to evaluate the association between the availability of fruits, vegetables, and ultra-processed beverages in the food environment and the presence of these conditions among children living in areas of social vulnerability.

## 2. Materials and Methods

### 2.1. Ethical Considerations

This study is part of a project conducted in accordance with the Declaration of Helsinki, and all procedures involving human subjects were approved by the Research Ethics Committee of the Federal University of Alagoas (approval number: 4.836.765). Written informed consent was obtained from all participants.

### 2.2. Study Design and Location

This is a cross-sectional (individual data) and partially ecological (environmental component) population-based study conducted between October 2020 and May 2021. The study aimed to assess the food environment, nutritional status, and other individual and socioeconomic characteristics of children living in favelas and urban communities in the Municipality of Maceió, the capital of the State of Alagoas, in the Northeast region of Brazil.

According to the Brazilian Institute of Geography and Statistics [20], favelas and urban communities are places with a predominance of households with varying degrees of legal insecurity of tenure and at least one of the other criteria: absence or incomplete provision of public services; predominance of buildings, streetscapes and infrastructure that are usually self-produced or are guided by urban planning and construction parameters other than those defined by public bodies; location in areas with restrictions on occupation defined by environmental or urban planning legislation.

### 2.3. Sample Size and Selection

To calculate the sample size, an estimated 11,430 children under 59 months of age residing in the 94 favelas and urban communities of Maceió were considered [21]. Based on this population, an overweight prevalence of 14.9% among children under 59 months of age in the state of Alagoas, Brazil, was used [22]. Adopting a margin of error of 3%, a 95% confidence level, and a 10% allowance to account for potential losses and refusals, it was estimated that at least 569 children aged 6 to 59 months would need to be recruited, distributed proportionally to the population size of each favela and urban community. The sample calculation was performed using StatCalc version 7.2.

**Figure 1:**
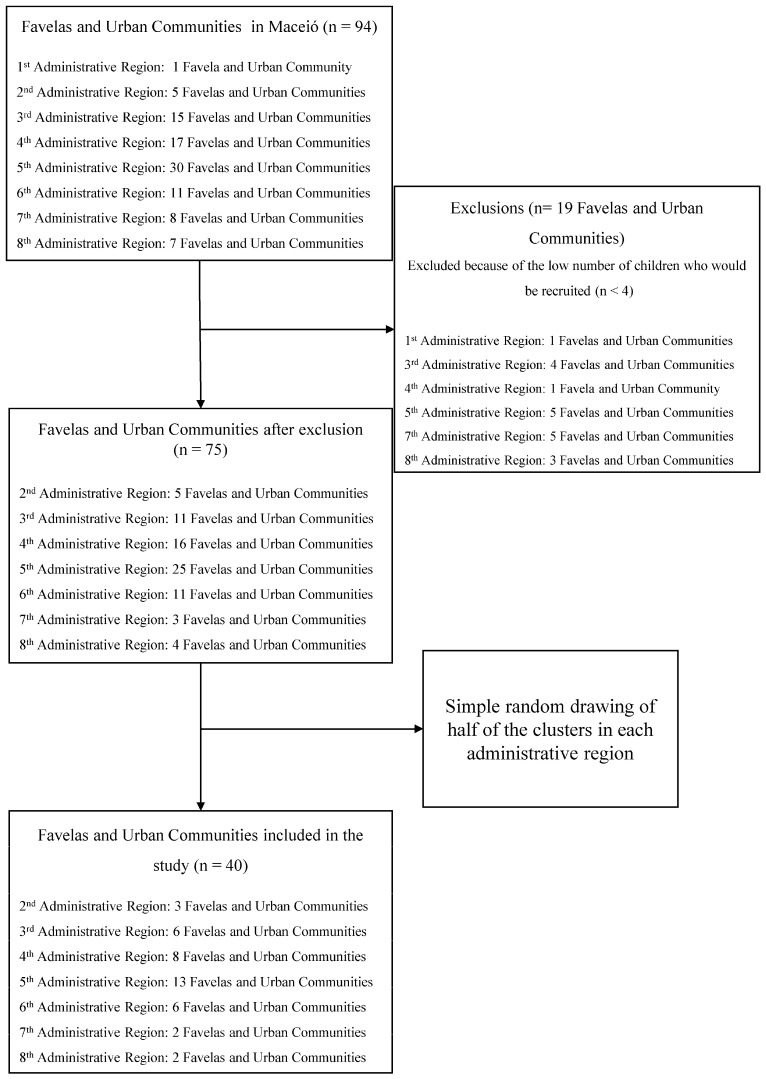
Flowchart of the selection of the subnormal settlements included in the study.

The sample was recruited from 40 favelas and urban communities. The selection process for the locations included in the study is presented in Figure 1. The sample was distributed proportionally to the number of inhabitants in each of the seven administrative regions evaluated.

The sampling design was a three-stage, cluster-based, probability sample: (1) selection of favelas and urban communities by simple random sampling, proportionally in each of the seven administrative regions studied; (2) selection of census tracts, with one tract selected by simple random sampling when the favela or urban community had more than one census tract; and (3) selection of the starting street for data collection, performed by random selection in each evaluated census tract.

All households on the selected street were visited, and whenever necessary, neighboring streets were also visited until the sample for that location was complete. All households with at least one child aged 6 to 59 months were included.

Children were excluded from the study if their mother had a disability that could prevent the interview from being conducted or the questionnaires from being understood, or if the child had a disability that compromised their food intake and/or the performance of the anthropometric assessment. In households with more than one child in the study age group, the youngest child was selected. When twins were present, a random selection was made before the interview began to determine which child would be included. Data were collected for only one child per household.

At the end of the recruitment process, 691 children were assessed, of whom 665 met the eligibility criteria and were included in the analyses. Detailed information on the recruitment, eligibility, and inclusion stages for participants can be found in the study by Silva-Neto et al. [5].

### 2.4. Data Collection

#### 2.4.1. Sociodemographic and Health Variables

The following information was collected about the sex (female/male), age (collected over several months and subsequently categorized as follows: ≤23 months; ≥24 months) of the children, number of children in the household, years of maternal schooling (<8 years; ≥8 years), and breastfeeding (yes/no). Per capita monthly family income was also assessed, classifying it according to the cut-off points for poverty (poverty—US$ < 95.59 and out of poverty US$ ≥ 95.59; values converted from reais to US dollars considering the dollar rate on 31 May 2021—R$ 5.22) [23].

#### 2.4.2. Anthropometric Assessment

Weight was measured using a digital scale (Avanutri^®^-Três Rios, Rio de Janeiro, Brazil), and height was measured using a portable infantometer and a stadiometer (Avanutri^®^-Três Rios, Rio de Janeiro, Brazil). The data were then entered into Anthro software version 3.2.2 to obtain the Z score values of the anthropometric indicator BMI for Age (BMI/A), classifying the children as thin (Z score < −2), eutrophic (Z score ≥ −2 and ≤+2), overweight (Z score > +2 and ≤+3), and obese (Z score > +3) [24]. For the regression analysis, the children were classified as follows: not overweight (BMI/A Z score ≤ +2) and overweight (BMI/A Z score > +2).

#### 2.4.3. Blood Tests to Check for Anemia

For anemia assessment, blood samples were collected via digital puncture to measure hemoglobin (Hb) concentration. A portable hemoglobinometer from HemoCue, the β Hemoglobin Photometer^®^ (Ängelholm, Sweden), was used, which employs photometric readings with microcuvettes containing β hemoglobin. Anemia was determined based on the Hb concentration in children (Children, 6–23 months Hb < 10.5 g/dL; Children, 24–59 months Hb < 11.0 g/dL) [25].

Of the 665 children included in the study, 403 underwent hemoglobin assessment. The absence of hemoglobin measurements among the remaining participants was due solely to a lack of maternal consent. It should be noted that all decisions regarding participation in this procedure were made voluntarily by the children’s legal guardians, and the research team did not exert any influence on the acceptance or refusal of participation.

#### 2.4.4. The Consumer’s Food Environment

All formal and informal retail establishments [26] found within a 400 m buffer from the midpoint of the randomly selected streets for data collection from children in each favela and urban community were audited. The 400 m buffer zone was adopted based on evidence indicating that this distance is strongly associated with children’s exposure to the food environment [27] and has been used previously in a study conducted in Brazil [28]. In addition, a 400 m radius corresponds approximately to a 5 min walk [29], representing a feasible distance for daily mobility and access to food outlets, particularly among children living in socially vulnerable contexts.

The total number of audited establishments was 624. The audit was conducted using the AUDITNOVA tool, which is validated for food retail in Brazil and assesses factors such as availability, price, variety, and advertising strategies in food retail [3].

Based on data collected during the audit, the presence of the following items related to fruits, vegetables, and greens was evaluated: zucchini, lettuce, banana, potato, onion, carrot, chayote, orange, apple, papaya, watermelon, and tomato. Additionally, the availability of ultra-processed beverages in retail stores was assessed. The following items were evaluated: fermented milk beverage with strawberry pulp (540 g packaging or with 6 small pots), tetrapack boxed nectar (1 L), powdered fruit drink, soft drinks (2 L), canned soft drinks (350 mL), and zero, light, or diet soft drinks (any size packaging).

Fruits, vegetables, and greens were selected as indicators of healthy food availability because their consumption has declined in recent years among children [17,30], particularly in low- and middle-income countries. In contrast, their availability remains limited in food-insecure settings [31,32]. On the other hand, ultra-processed beverages were selected as indicators of unhealthy food availability due to their widespread presence in food retail establishments [33] and their growing consumption, especially among children and socially vulnerable populations [34].

#### 2.4.5. Standardization of Food Environment Components

Food environment variables were rescaled to improve the interpretability of the regression coefficients and facilitate comparisons across exposures. Because the original units represented very small changes in availability, analyses based on single-unit increments could produce effect estimates of limited practical relevance.

Therefore, the availability of fruits, vegetables, and greens, as well as ultra-processed beverages, was divided by five before modeling. As a result, the estimated odds ratios represent the change in the outcome associated with each increase of five units in the availability of the respective food environment component.

#### 2.4.6. Spatial Data

The geographic coordinates (latitude and longitude) of all audited retail establishments were collected using the Google Earth application v. 9.3.25.5 (Google LLC, Mountain View, CA, USA), positioned at a distance of 1 m from their main entrance. Subsequently, this data was input into the QGIS 3.16.15 software (Open Source Geospatial Foundation, Chicago, IL, USA).

A buffer layer with a radius of 400 m was created, calculated from the midpoint of each randomly selected street for data collection in each favela and urban community. After overlaying this layer with another layer containing establishment-level data, the average values of fruit, vegetable, and greens availability, as well as ultra-processed beverages, were calculated for each buffer. This provided information for each favela and urban community.

#### 2.4.7. Data Analysis

Descriptive analyses of individual characteristics were conducted. Variables describing the food environment were analyzed continuously and presented with median, maximum, minimum, and interquartile ranges (IQR). Excess weight (BMI/A Z-score > +2) and anemia (Hb < 11 g/dL) were considered as outcome variables. The assumptions of normality were assessed using the Kolmogorov–Smirnov test with Lilliefors correction.

Because the exposure variables were measured at the community level and the outcomes at the individual level, multilevel models were used to account for the hierarchical structure of the data and to estimate associations between contextual exposures and individual health outcomes appropriately.

Multilevel logistic regression models were conducted within a multilevel analytical structure at two hierarchical levels: favelas and urban communities and children. Initially, null models containing only the random intercept were fitted to estimate between-community variability and the intraclass correlation coefficient (ICC) for excess weight and anemia. Subsequently, these models assessed the influence of food availability in the food environment (fruits, vegetables, and greens; ultra-processed beverages) on malnutrition-related outcomes (excess weight and anemia). The multilevel framework was adopted because exposure variables were measured at the community level, whereas outcomes were measured at the individual level, thereby accounting for the hierarchical structure of the data. In this analysis, probabilities were calculated using odds ratios (OR) along with their respective 95% confidence intervals (95% CI).

The modeling procedure was conducted in a series of steps, with the availabilities of the assessed foods included as continuous variables. Model 1 included the availability of fruits, vegetables, and greens—the values were obtained as the average number of assessed foods available in each favela and urban community. Model 2 included the availability of ultra-processed beverages—the values were obtained as the average number of assessed beverages available in each favela and urban community. The models that included excess weight as an outcome were adjusted for individual variables (age, sex, years of maternal schooling, and breastfeeding) and for socioeconomic variables (family per capita income). The models that included anemia as an outcome were adjusted for individual variables (age, sex, years of maternal schooling, breastfeeding, and excess weight) and for socioeconomic variables (family per capita income).

The categorization of variables in the modeling process was tested in different forms (continuous, percentiles, and tertiles), and the one that best fits the models was selected. The Akaike Information Criterion (AIC) was used to compare models during the sensitivity analysis. The analyses were conducted using the statistical software R v. 4.6.0 (R Foundation for Statistical Computing, Vienna, Austria) with the “lme4” package. A significance level of 5% was adopted.

## 3. Results

Among the 665 children evaluated, 54.1% were male, and 69.3% were aged 24 months or older (Table 1). In addition, 91% were living in poverty. Regarding nutritional status, 19.7% were overweight, and 50.4% had anemia (Table 1). An average of 2.21 (SD 1.10) children was also identified in the households evaluated.

Food availability in the food environment is presented in Table 2. Among fruits and vegetables, the items most commonly available in retail outlets were onions (32.4%), tomatoes (31.7%), potatoes (30.4%), and bananas (29.5%). Regarding ultra-processed beverages, soft drinks (73.1%) and powdered drink mixes (68.9%) were the most prevalent items.

In the multilevel analysis, the null models showed significant variability across communities. The ICC indicated that 7.1% of the variability in overweight status and 13.2% of the variability in anemia were attributable to differences between communities. These findings supported the use of multilevel models to account for the hierarchical structure of the data.

The association between environmental food availability and the presence of overweight and anemia in children, estimated using multilevel logistic regression models, is presented in Table 3. In the adjusted analysis, each increase of five units in the availability of fruits and vegetables was associated with a 18% lower chance of overweight among children (OR: 0.82; 95%CI: 0.79–0.98) (Model 1). In Model 2, each increase of five units in the availability of ultra-processed beverages was associated with a 35% higher chance of overweight (OR: 1.35; 95%CI: 1.07–1.84). For anemia, each increase of five units in the availability of ultra-processed beverages was associated with a 53% higher chance of the outcome (OR: 1.53; 95%CI: 1.04–2.29).

Fruit, vegetables, and greens evaluated: zucchini, lettuce, banana, potato, onion, carrot, chayote, orange, apple, papaya, watermelon, and tomato;

Ultra-processed beverages evaluated: fermented milk beverage with strawberry pulp (540 g packaging or with 6 small pots), tetrapack boxed nectar (1 L), powdered fruit drink, soft drinks (2 L), canned soft drinks (350 mL), and zero, light, or diet soft drinks (any size packaging).

## 4. Discussion

This study demonstrates that, in areas of social vulnerability, the availability of ultra-processed beverages exceeds that of fresh foods, creating a food environment unfavorable to children’s health. Our findings reinforce the relevance of the food environment as a determinant of nutritional status, especially among more vulnerable populations.

Among the main findings, we highlight that each increase of five units in the availability of ultra-processed beverages in the food environment was associated with a 35% and 53% higher chance of overweight and anemia among children, respectively. It was also possible to identify that each increase of five units in the availability of fruits and vegetables was associated with a 18% lower chance of childhood overweight.

The consumer food environment identified in this study aligns with the concept of a “food swamp,” recognized as conducive to obesity [35], as the predominance of UPFs, especially ultra-processed beverages, may favor unhealthy food choices.

This situation is aggravated by the fact that most families live below the poverty line, in agreement with data from the United Nations Children’s Fund indicating the low economic capacity of Brazilian families. It is estimated that approximately 60% of children in the North and Northeast regions of Brazil live on less than US$5.50 per day [36]. This context limits the prospects for positive advances in nutrition by combining restricted financial resources for purchasing healthy foods with an environment characterized by the abundant availability of UPFs.

In this context, the dominant food system and the corresponding food environment may influence food choices and dietary behaviors, which have been associated with excessive weight gain in the population. This situation is evident during complementary feeding, a critical stage for establishing healthy eating habits [37]. As children grow, continuous exposure to and wide availability of unhealthy foods with excessive energy and sugar content, such as ultra-processed beverages, raise concerns regarding the dietary choices made by this population [38].

The greater availability of ultra-processed beverages identified in this study reinforces the growing body of evidence regarding the food environment across different regions of Brazil, where the commercialization of UPFs has expanded significantly in recent decades [32,39], particularly ultra-processed beverages [40]. Similar situations have been observed in Mozambique, where 59% of the evaluated establishments offered UPFs [41], demonstrating the widespread availability of these products in low- and middle-income countries.

This scenario, characterized by greater availability of UPFs in lower-income regions, may be one of several factors related to the 19.7% prevalence of overweight observed in this study. However, comparisons with national estimates should be interpreted cautiously, as the children included in the present investigation lived in conditions of marked social vulnerability, which differ substantially from those of the general Brazilian population. These findings reinforce that more vulnerable populations are strongly affected by environmental factors, such as the high availability of UPFs, which directly influence the formation of dietary habits during childhood [42]. The concern becomes even greater given that the introduction of UPFs has occurred earlier among children from low-income families in Brazil [5,7].

In contexts of greater socioeconomic vulnerability, the high prevalence of these products in retail establishments near households may favor their acquisition and consumption, potentially influencing diet quality [33,43]. This reality may be associated with lower consumption of vegetables and other healthy foods and a greater preference for products with lower nutritional value, such as ultra-processed beverages [44].

Furthermore, previous studies have shown that higher consumption of UPFs is associated with lower intake of micronutrients essential to health, such as iron [45,46]. Although the present study evaluated food availability rather than dietary intake, the greater availability of ultra-processed beverages in the food environment may reflect conditions less favorable to healthy dietary practices. In this context, iron deficiency is one of the main causes of childhood anemia [47], which may help explain the relationship between the high prevalence of anemia found in this study (50.4%), approximately five times higher than the Brazilian average (10.3%) [48], and the greater availability of ultra-processed beverages in the consumer food environment, also identified in this study.

Although a positive association was observed between the availability of ultra-processed beverages and anemia, this finding should be interpreted cautiously and considered alongside evidence from other studies, particularly given the multifactorial nature of anemia and the cross-sectional design of the present investigation. In addition to dietary factors, anemia may be influenced by infections, parasitic diseases, nutritional deficiencies, and other social and environmental determinants. Furthermore, studies directly examining associations between food availability in local food environments and childhood anemia remain limited.

Our findings also indicate that greater availability of fruits and vegetables is associated with a lower chance of overweight among children. When the environment in which children live offers a greater supply of healthy foods such as these, greater opportunities for access and consumption may be created. However, dietary intake was not assessed in the present study. At the same time, prices may decrease due to greater availability and the resulting demand. Consuming fruits and vegetables is associated with increased satiety and may help prevent overweight and obesity [49], corroborating our findings.

Children’s exposure to a food environment with greater availability of fresh foods may favor healthier dietary patterns, although this relationship was not directly evaluated in the present investigation. This scenario is associated with excessive weight gain and increased prevalence of non-communicable chronic diseases, especially among younger populations [50]. Therefore, the need for initiatives that facilitate the population’s access to fruits and vegetables, particularly in more vulnerable regions, becomes evident to improve the food environment [51].

It is also observed that greater consumption of UPFs is associated with lower dietary iron availability and a greater chance of anemia [52]. However, because the present study assessed food availability rather than food consumption, it is not possible to determine whether children exposed to environments with greater availability of UPFs actually consumed these products more frequently. Thus, greater availability of UPFs in the food environment may represent a context that is less conducive to healthy eating practices, including the regular consumption of fruits and animal-source foods [53]. This situation is particularly concerning because animal-source foods are essential for meeting the body’s iron requirements and for adopting healthy eating habits [54].

The findings of this study reaffirm the need to discuss the influence of the food environment, especially for individuals living in situations of social vulnerability. These population groups are more exposed to the effects of their environment’s characteristics and face limited opportunities for change due to income constraints. Therefore, they must be inserted into a food environment that promotes the development of healthy eating habits, a protective factor for weight maintenance and obesity control [51].

This study has limitations. The cross-sectional design reduces the ability to establish temporal relationships and causal inferences. Reverse causality cannot be ruled out, and the observed associations should be interpreted as contextual relationships rather than causal effects. In addition, because food environment measures were assessed at the community level, whereas outcomes were measured at the individual level, findings should be interpreted in conjunction with other evidence on childhood nutritional outcomes. Another limitation is that hemoglobin measurements were obtained for only a subset of participants, which may have introduced selection bias, although participation depended exclusively on parental consent. Also, because the 400 m catchment area provides a standardized measure of local dietary exposure, individual exposure may vary depending on daily mobility patterns, food-purchasing practices, and the use of food establishments located outside the assessed area. Furthermore, changes in the location and sales profile of establishments may occur over time.

Among the study’s strengths, we highlight its potential to reinforce the importance of environmental determinants in the various forms of childhood malnutrition, especially among populations living in situations of social vulnerability and below the poverty line. It is also important to emphasize that the assessment of the food environment was conducted through an audit of all commercial establishments within the evaluated areas.

## 5. Conclusions

In conclusion, overweight among children was associated with greater availability of ultra-processed beverages in the food environment. In contrast, greater availability of fruits and vegetables was associated with a lower chance of being overweight. In addition, greater availability of ultra-processed beverages was also associated with a higher chance of anemia among children. These findings highlight the coexistence of food environment characteristics and nutritional outcomes among children living in situations of social vulnerability and support the development of strategies to promote healthier, more equitable food environments.

## Figures and Tables

**Table 1:** Child, maternal, and household characteristics in favelas and urban communities in the municipality of Maceió-Alagoas, Northeast Brazil, 2020/2021 (*n* = 665).

Variables	*n*	%
Gender		
Female	305	45.9
Male	360	54.1
Age		
≤23 months	204	30.7
≥24 months	461	69.3
Years of maternal schooling *		
<8 years	308	46.3
≥8 years	357	53.7
Breastfeeding ^#^		
No	44	6.6
Yes	621	93.4
Poverty status ^¥^		
No	60	9.0
Yes	605	91.0
Nutritional status ^€^		
Thinness	24	3.6
Eutrophic	510	76.7
Overweight	76	11.4
Obesity	55	8.3
Anemia ^£^		
No	200	49.6
Yes	203	50.4

* <8 years: corresponds to incomplete elementary school; ≥8 years: corresponds to at least a complete elementary school education. ^#^ This variable represented any history of breastfeeding, regardless of its duration or whether the child may have been exclusively breastfed at any stage. ^¥^ Classified it according to the cut-off points for poverty (poverty—US$ < 95.59 and out of poverty US$ ≥ 95.59; values converted from reais to US dollars considering the dollar rate on 31 May 2021—R$ 5.22) (World Bank, 2022 [23]). ^€^ Classified according to z-score values (Overweight—BMI for Age > +2). ^£^ Of the 665 children included in the study, 403 underwent hemoglobin assessment. The absence of hemoglobin measurements among the remaining participants was due solely to a lack of maternal consent. Classified based on the Hb concentration in children (Children, 6–23 months Hb < 10.5 g/dL; Children, 24–59 months Hb < 11.0 g/dL) (WHO, 2024 [25]).

**Table 2:** Characterization of the food environment, according to the number of establishments that had each food assessed (*n* = 624).

Food	*n*	%
Fruits, vegetables, and greens		
Onion	202	32.4
Tomato	198	31.7
Orange	134	21.5
Banana	184	29.5
Papaya	134	21.5
Apple	118	18.9
Watermelon	136	21.8
Lettuce	98	15.7
Carrot	170	27.2
Zucchini	44	7.1
Chayote	154	24.7
Potato	190	30.4
Ultra-processed beverages		
Fermented milk beverage with strawberry pulp	154	24.7
Tetrapack boxed nectar	66	10.6
Powdered fruit drink	430	68.9
Soft drinks	456	73.1
Canned soft drinks	318	51.0
Zero, light, or diet soft drinks	102	16.3
	Median (IQR)	Min–Max
Fruits and vegetables	3.1 (2.4–3.8)	2.4–9.6
Ultra-processed beverages	2.3 (2.0–2.5)	1.0–4.0

IQR: interquartile range.

**Table 3:** Associations between the availability of ultra-processed beverages and fruits and vegetables in the food environment and the presence of overweight and anemia in socially vulnerable Brazilian children, using multilevel logistic regression models (2020–2021).

		Model 1 *			Model 2 *	
	OR	95% CI	*p*-Value	OR	95% CI	*p*-Value
Overweight (*n* = 665)						
Fruits, legumes, and vegetables	0.82	[0.79; 0.98]	0.010			
Ultra-processed beverages				1.35	[1.07; 1.84]	0.031
Corrected Akaike (AICc)		523.12			512.10	
Precision		59.9%			61.4%	
Anemia (*n* = 403)						
Fruits, legumes, and vegetables	0.89	[0.77; 1.21]	0.253			
Ultra-processed beverages				1.53	[1.04; 2.29]	0.029
Corrected Akaike (AICc)		356.39			338.27	
Precision		61.8%			63.9%	

OR, odds ratio; 95% CI, 95% confidence interval. Model 1 included the availability of fruits, vegetables, and greens—the values were obtained using the average number of assessed foods available in each favela and urban community. Model 2 included the availability of ultra-processed beverages—the values were obtained using the average number of assessed beverages available in each favela and urban community. * The models that had excess weight as an outcome were adjusted for individual variables (age, sex, years of maternal schooling, and breastfeeding) and socioeconomic variables (family per capita income). The models that had anemia as an outcome were adjusted for individual variables (age, sex, years of maternal schooling, breastfeeding, and excess weight) and socioeconomic variables (family per capita income).

## Data Availability

The datasets generated and/or analyzed during the current study are not publicly available because they are part of ongoing doctoral and master’s research projects. Data may be made available by the corresponding author upon reasonable request after completion of the related studies.
